# Supplementary material for: Investigating workplace bullying (WPB), intention to quit and depression among nurses in the Upper West Region of Ghana
Source: PLoS One. 2024 Nov 25;19(11):e0305026. doi: 10.1371/journal.pone.0305026 (PMC11588229; doi:10.1371/journal.pone.0305026)
Supplement: S1 File — (DOCX) [file pone.0305026.s001.docx]

**S1 APPENDIX A**

**Data Collection Instrument**

STRUCTURE OF THE QUESTIONNAIRE

UNIVERSITY OF CAPE COAST

SCHOOL OF NURSING AND MIDWIFERY

DEPARTMENT OF ADULT HEALTH

Research Instrument@2020

**Background**

Dear Respondent,

My name is Emmanuel Dapilah, a Registered General Nurse (RGN) and Master of Nursing (MN) student at the School of Nursing and Midwifery, University of Cape Coast (UCC). I am conducting research on the ***workplace bullying, depression, and intention to quit among nurses in the*** ***Upper West Region****”* as part of the requirements for the award of the Master of Nursing Degree. This study will determine the magnitude of bullying and create awareness of the existence of the phenomenon in our health facilities. This will serve as the starting point for the formulation and implementation of policies by stakeholders and managements aimed at either preventing or reducing it in the workplace. Your participation in this study is voluntary and will mean completing a questionnaire which will take 10-15 minutes of your time. You may also pull out of the study at any time during the study without any punitive actions against you. Your responses are completely anonymous and be assured that the information you give will be kept confidentially. If you have any questions about this survey, please email me at [emmanuel.dapilah@stu.ucc.edu.gh](mailto:emmanuel.dapilah@stu.ucc.edu.gh) or call me on 0200955210/0241356185. Thank you for your decision to take part in this study.

**PLEASE ANSWER THE FOLLOWING QUESTIONS AS OBJECTIVELY AS POSSIBLE BY TICKING (√) THE RESPONSES THAT APPLY IN EACH CASE OR WRITING DOWN THE ANSWERS IN THE SPACES PROVIDED**

**SECTIONS A: DEMOGRAPHIC CHARACTERISTICS OF RESPONDENTS**

1. Gender:
2. Male
3. Female
4. What is your age? (*please specify*) ..............................................................................years
5. Please indicate your professional nursing background
6. Enrolled Nurse
7. Registered General Nurse
8. What is your current rank (position) in your profession? (*please specify*) ..............................

**SECTION B: INTENTIONS TO QUIT**

1. Have you considered quitting your present job over the last six (6) months?
   1. Never
   2. Rarely
   3. Sometimes
   4. Quite often
   5. Very often

**SECTION C: PREVALENCE OF WORKPLACE BULLYING AMONG NURSES**

***The following behaviours are often seen as examples of negative behaviour in the workplace*. *Over the last six months, please indicate how often have you been subjected to the following negative acts at work. Use the key in the first table to guide you.***

***Please circle the number that best corresponds with your experience over the last six months:***

| 1 | 2 | 3 | 4 | 5 |
| --- | --- | --- | --- | --- |
| Never | Now and then | Monthly | Weekly | Daily |

| 1) Someone withholding information which affects your performance | 1 2 3 4 5 |
| --- | --- |
| 2) Being humiliated or ridiculed in connection with your work | 1 2 3 4 5 |
| 3) Being ordered to do work below your level of competence | 1 2 3 4 5 |
| 4) Having key areas of responsibility removed or replaced with more trivial or unpleasant tasks | 1 2 3 4 5 |
| 5) Spreading of gossip and rumours about you | 1 2 3 4 5 |
| 6) Being ignored or excluded (being ‘sent to Coventry’) | 1 2 3 4 5 |
| 7) Having insulting or offensive remarks made about your person (i.e. habits and background), your attitudes or your private life | 1 2 3 4 5 |
| 8) Being shouted at or being the target of spontaneous anger (or rage) | 1 2 3 4 5 |
| 9) Intimidating behaviour such as finger-pointing, invasion of personal space, shoving, blocking/barring the way | 1 2 3 4 5 |
| 10) Hints or signals from others that you should quit your job | 1 2 3 4 5 |
| 11) Repeated reminders of your errors or mistakes | 1 2 3 4 5 |
| 12) Being ignored or facing a hostile reaction when you approach | 1 2 3 4 5 |
| 13) Persistent criticism of your work and effort | 1 2 3 4 5 |
| 14) Having your opinions and views ignored | 1 2 3 4 5 |
| 15) Practical jokes carried out by people you don’t get on with | 1 2 3 4 5 |
| 16) Being given tasks with unreasonable or impossible targets or deadlines | 1 2 3 4 5 |
| 17) Having allegations made against you | 1 2 3 4 5 |
| 18) Excessive monitoring of your work | 1 2 3 4 5 |
| 19) Pressure not to claim something which by right you are entitled to (e.g. sick leave, holiday entitlement, travel expenses) | 1 2 3 4 5 |
| 20) Being the subject of excessive teasing and sarcasm | 1 2 3 4 5 |
| 21) Being exposed to an unmanageable workload | 1 2 3 4 5 |
| 22) Threats of violence or physical abuse or actual abuse | 1 2 3 4 5 |

***23. Have you been bullied at work? We define bullying as a situation where one or several individuals persistently over a period of time perceive themselves to be on the receiving end of negative actions from one or several persons, in a situation where the target of bullying has difficulty in defending him or herself against these actions. We will not refer to a one-off incident as bullying*.**

Using the above definition, please state whether you have been bullied at work over the last six months?

No

Yes, but only rarely

Yes, now and then

Yes, several times per week

Yes, almost daily

25. If your answer to the previous question was “**Yes**”, please tick the appropriate box(es) below to state who you were bullied by:

My immediate superior

Other superiors/managers in the organisation

Colleagues

Subordinates

Customers/patients/students, etc.

Others

25. Please state the number and gender of your perpetrators:

Male perpetrators

Female perpetrators

**SECTION D: LEVEL OF DEPRESSION AMONG NURSES**

***The statements below are on the level of depression among nurses. Please indicate how much the statement applied to you over the past weeks by circling a number; 0, 1, 2 or 3. There are no right or wrong answers. Do not spend too much time on any statement. Use the rating scale below to guide you***

The rating scale:

**0** Did not apply to me at all-NEVER (N)

**1** Applied to me to some degree, or some of the time-SOMETIMES (S)

**2** Applied to me to a considerable degree or a good part of time-OFTEN (O)

**3** Applied to me very much or most of the time-ALMOST ALWAYS (AA)

|  |  | **N** | **S** | **O** | **AA** |
| --- | --- | --- | --- | --- | --- |
| 1 (s) | I found it hard to wind down | 0 | 1 | 2 | 3 |
| 2 (a) | I was aware of dryness of my mouth | 0 | 1 | 2 | 3 |
| 3 (d) | I couldn’t seem to experience any positive feeling at all | 0 | 1 | 2 | 3 |
| 4 (a) | I experienced breathing difficulty (e.g. excessively rapid breathing, breathlessness in the absence of physical exertion) | 0 | 1 | 2 | 3 |
| 5 (d) | I found it difficult to work up the initiative to do things | 0 | 1 | 2 | 3 |
| 6 (s) | I tended to over-react to situations | 0 | 1 | 2 | 3 |
| 7 (a) | I experienced trembling (e.g. in the hands) | 0 | 1 | 2 | 3 |
| 8 (s) | I felt that I was using a lot of nervous energy | 0 | 1 | 2 | 3 |
| 9 (a) | I was worried about situations in which I might panic and make a fool of myself | 0 | 1 | 2 | 3 |
| 10 (d) | I felt that I had nothing to look forward to | 0 | 1 | 2 | 3 |
| 11 (s) | I found myself getting agitated | 0 | 1 | 2 | 3 |
| 12 (s) | I found it difficult to relax | 0 | 1 | 2 | 3 |
| 13 (d) | I felt down-hearted and blue | 0 | 1 | 2 | 3 |
| 14 (s) | I was intolerant of anything that kept me from getting on with what I was doing | 0 | 1 | 2 | 3 |
| 15 (a) | I felt I was close to panic | 0 | 1 | 2 | 3 |
| 16 (d) | I was unable to become enthusiastic about anything | 0 | 1 | 2 | 3 |
| 17 (d) | I felt I wasn’t worth much as a person | 0 | 1 | 2 | 3 |
| 18 (s) | I felt that I was rather touchy | 0 | 1 | 2 | 3 |
| 19 (a) | I was aware of the action of my heart in the absence of physical exertion (e.g. sense of heart rate increase, heart missing a beat) | 0 | 1 | 2 | 3 |
| 20 (a) | I felt scared without any good reason | 0 | 1 | 2 | 3 |
| 21 (d) | I felt that life was meaningless | 0 | 1 | 2 | 3 |
