# Supplementary material for: Investigating workplace bullying (WPB), intention to quit and depression among nurses in the Upper West Region of Ghana
Source: PLoS One. 2024 Nov 25;19(11):e0305026. doi: 10.1371/journal.pone.0305026 (PMC11588229; doi:10.1371/journal.pone.0305026)
Supplement: Attachment — Submitted filename: Response to reviewers.docx [file pone.0305026.s003.docx]

**Response to reviewers’ comments**

Reviewer #1: Dear authors,

Thank you for providing me with the opportunity to review this manuscript. It is well-written. However, there are some minor changes that I recommend making:

1. In the abstract, you mentioned using a multistage sampling technique, but in the methods section, you stated that a cluster sampling technique was used. Please ensure consistency in your approach.

Response: Thank you for this observation. We have edited this portion to reflect the exact sampling technique i.e. stratified sampling and ensured its consistent usage across the manuscript.

2. In the data analysis section, you mentioned that the analysis was conducted to address research questions and hypotheses. However, there were no research questions or hypotheses presented in the manuscript.

Response: we meant the analysis was done to address the purpose of the study. We have revised the manuscript to correct this. Thank you.

3. Many studies cited in the literature review are outdated. Please provide justification for using older studies or update the literature review with more recent studies.

Response: we have replaced some of the old references where possible with current ones.

Thank you for your attention to these suggestions.

Reviewer #2: I have read with interest the manuscript titled ‘Investigating Workplace Bullying, Intention to Quit and Depression Among Nurses in the Upper West Region of Ghana’, which presents a correlational study testing the relationship between workplace bullying and turnover intention and depression among nurses in Ghana.
I appreciated the rationales behind the research, indeed, replicating previous finding and extending them to diverse social and cultural contexts is important; however, I have some concerns about the current form of the manuscript, which I will outline below.

My concerns are mainly focused on the rationales to include/exclude factors in the major statistical analyses and their theoretical importance. Indeed, I generally found that in their design and data analyses authors took into consideration some factors (e.g., registered general nurses vs. enrolled nurses, gender, age groups, rank) without these factors being necessarily introduced in the theoretical introduction. It is unclear what the literature says about the importance of these factors in the relationship between bullying and intentions to quit work and or depression among nurses.

So, I found an inconsistency between some analyses (e.g., multiple linear regression) and theoretical background.

I suggest that the authors explain in the theoretical introduction why these factors are important and explain why they were taken into consideration based on the literature of reference.
As an example, as a reader I was (and still I am) unaware about the difference between registered general nurses and enrolled nurses. I think it is necessary to explain what the difference is and why this factor was taken into consideration. Is it important for the relationship between workplace bullying and the outcome variables? Or do the authors considered it in an exploratory fashion. The same rationales apply to age group, gender and rank position. Moreover, why the authors decided to collapse those specific age groups? What was the rationale? Why not to include age as a continuous variable if age is considered an important factor?

Response: we appreciate your concerns. We have included some literature regarding the demographic factors. Though the demographic variables may play significant role in the relationship between WPB, depression, and intention to quit, they are not the primary factors in this study and only served as covariates. Also, the variable age was categorized into groups to ascertain if certain age groups will be more likely to be affected than others. For example, the literature shows that younger nurses and junior nurses have a higher propensity to leave the profession and to be bullied.

Another point, on page 6 of the manuscript, the authors indicate that the questionnaire used can be found in Appendix A. However, downloading the supplementary materials, one does not find the questionnaire with the measures used, but other materials (e.g., Ethical clearance and Consent Form). While I found that, in the section describing the measures, there were enough information about how depression and intention to quit work were present, I suggest the authors to provide more information on the measure of workplace bullying. For example, does the NAQ-R tap into a single dimension or into several ones? If it has subscales, which dimensions they represents and why they were collapse into a single variable as specified at page 9 of the current manuscript. Also, I found it would be interesting for the reader to have examples of items if the questionnaire is not available as an appendix.

Response: we have realized we did not attach the questionnaire as an appendix as indicated in the manuscript. Hence, we have attached the questionnaire in this revision as S1 Appendix A. Also, we have included adequate information on the measure of workplace bullying as suggested. Thank you for your input.
